# Simplified synthesis of oxidized phospholipids on alkyl-amide scaffold

**DOI:** 10.1016/j.mex.2025.103288

**Published:** 2025-03-27

**Authors:** Olga V. Oskolkova, Bernd Gesslbauer, Valery Bochkov

**Affiliations:** a Institute of Pharmaceutical Sciences, Division of Pharmaceutical Chemistry, University of Graz, Humboldtstrasse 46/III, Graz 8010, Austria; b Field of Excellence BioHealth, University of Graz, Graz, Austria

**Keywords:** Oxidized phospholipids, Synthesis, Oxylipins, Synthesis of alkyl-amide OxPLs

## Abstract

Oxidized phospholipids (OxPLs), containing oxidized fatty acids (oxylipins), play a significant role in various diseases. However, studying the structure-activity relationships of OxPLs and their signaling mechanisms is challenging due to the complexity of the chemical synthesis of structurally distinct lipid species. In this study, we aimed to develop a method for attaching free oxylipins to a lysophospholipid to form OxPLs. We hypothesized that oxylipins could be conjugated to PLs *via* a known chemical reaction between activated esters of carboxylic acids and amino groups. The carboxyl groups of oxylipins were activated using N-hydroxysuccinimide and a coupling reagent, then conjugated to a lyso-phosphatidylcholine containing NH_2_-groupd at *sn*-2 position, forming amide bonds. All reactions were performed under mild conditions and demonstrated high yields. To prevent acyl migration, the *sn*-1 position of PLs was modified with an alkyl residue linked *via* an ether bond. Several oxylipin-containing PLs were successfully synthesized, isolated, and characterized. The anti-TLR4 and endothelial barrier-protective activities of these alkyl-amide OxPLs were found to be equivalent to diacyl-OxPLs. This method enables efficient synthesis of modified OxPLs for biological testing. The combination of ether and amide bonds enhances biological stability and simplifies effect analysis.

• The method describes the preparation of a single precursor for multiple choline PLs, specifically 2-deoxy-2-amino-1-lyso-*sn*-3-glycerophosphocholine, followed by the attachment of oxylipins to it.
• No protection-deprotection steps are needed for oxylipins for the synthesis of phosphatidylcholines.
• Isolation of compounds is performed using fast liquid-liquid and solid-phase extractions.

## Graphical abstract

**Figure:**
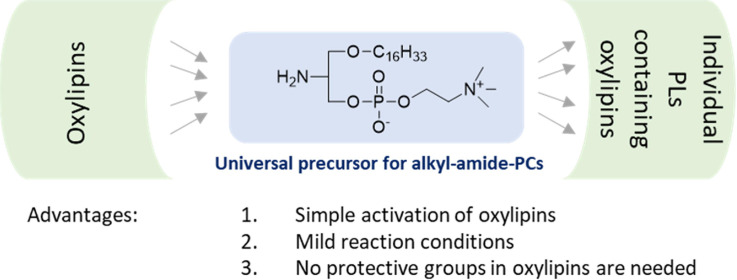

Specifications table

**Table:** 

Subject area:	Pharmacology, Toxicology and Pharmaceutical Science
More specific subject area:	Chemical synthesis of oxidized phospholipids
Name of your method:	Synthesis of alkyl-amide OxPLs
Name and reference of original method:	Oskolkova, O.V., Hodzic, A., Karki, P., Gesslbauer, B., Ke, Y., Hofer, D.C., Bogner-Strauss, J.G., Galano, J.M., Oger, C., Birukova, A., Durand, T., Birukov, K., Bochkov, V, Oxidized phospholipids on alkyl-amide scaffold demonstrate anti-endotoxin and endothelial barrier-protective properties, Free Radic Biol Med. 174 (2021) 264–271. 10.1016/j.freeradbiomed.2021.07.041
Resource availability:	All reagents and methods are described in the manuscript

## Background

Oxidized phospholipids (OxPLs) are products of enzymatic or non-enzymatic oxidation of polyunsaturated fatty acids bound to phospholipids [1,2]. Increasingly recognized as pathogenic factors in different clinical conditions [3,4], OxPLs can be classified into two structural groups: short-chain OxPLs and long-chain OxPLs [1]. The short-chain group is characterized by oxidatively truncated fatty acid residues, while the long-chain group contains oxidized fatty acids of the same carbon chain length as the parent (unoxidized) phospholipid.

Short-chain and long-chain OxPLs exhibit overlapping but not identical biological activities. Truncated OxPLs are toxic [5], pro-inflammatory [6], and pro-thrombogenic [7]. They act as weak antagonists of TLR4-induced inflammation [8] and disrupt the endothelial barrier in lung vessels, leading to edema [9]. In contrast, long-chain OxPLs are also pro-inflammatory [8] and pro-thrombogenic [10], but they act as stronger antagonists of TLR4 signaling, effectively inhibiting lipopolysaccharide-induced inflammation [8], and enhance the lung endothelial barrier [11]. The majority of biological activities of OxPLs were studied using complex mixtures of molecular species generated by non-enzymatic oxidation, such as the oxidized 1-palmitoyl-2-arachidonoyl-*sn*-3‑glycero-phosphocholine. The overall effect of an OxPL mixture depends on the ratio of different species and the inflammatory context [12]. Therefore, pure individual OxPL species are essential for a more detailed investigation of the biological roles of specific OxPLs. While the chemical synthesis of individual short-chain OxPLs has been described in several publications [13,14], the synthesis of long-chain OxPLs presents greater challenges due to the presence of variable functional groups in the acyl residues. Hydroxy-functional groups in oxylipins must be protected before coupling to a lyso-PL and later deprotected, which requires additional effort and reduces product yields. As a result, only a few representatives of long-chain OxPLs have been synthesized, such as epoxyisoprostanoyl-E2-phosphatidylcholine [15,16], isoprostanoyl-phosphatidylcholine and -ethanolamine [17], or deoxy-Δ12,14-prostaglandin J2-phosphatidylcholine [18].

Within this work, a method was developed for the synthesis of long-chain oxidized phosphatidylcholines on an alkyl-amide scaffold [19]. The final PLs have an alkyl residue at the *sn*-1 position linked to a glycerol backbone *via* an ether bond and an oxidized fatty acid residue linked to the *sn*-2 position *via* an amide bond. The combination of ether and amide bonds in one PL molecule (i) prevents acyl migration, which usually takes place during the synthesis of diacylglycero-PLs, and (ii) makes the compound resistant to phospholipases A1/A2, which are unable to cleave ether or amide bonds. Our method eliminates the need for protection-deprotection of functional groups in oxylipins and uses fast purification procedures including liquid-liquid and solid-phase extractions producing OxPLs in milligram amounts sufficient for experiments in cell culture or *in vivo* studies in mice.

## Method details

### Materials

12-Hydroxydodecanoic acid, 8-hydroxyoctanoic acid, *N*-hydroxysuccinimide, water-free tetrahydrofuran (THF) supplemented with butylated hydroxy toluene, water-free dichloromethane (≤0.001 % water), di‑*tert*‑butyl dicarbonate (Boc anhydride), 0.45 M 1*H*-tetrazole solution in acetonitrile, *tert*‑butyl hydroperoxide (∼5.5 M in decane), triethylamine (TEA), H_3_PO_4,_ and DSC—C18 SPE columns (100 mg, 1 ml) were purchased from Sigma-Aldrich (St. Louis, MO, USA). 1-Ethyl-3-(3-dimethylaminopropyl)carbodiimide hydrochloride (EDC), choline tosylate, and CuSO_4_ were from Thermo Fisher Scientific (Waltham, MA, USA). Diisopopylammonium tetrazolide was prepared by adding of 10-fold molar excess of diisopropylamine to 1*H*-tetrazol, followed by removal of the diisopropylamine on a rotary evaporator. Prostaglandins and isoprostanes were purchased from Cayman Chemicals (Ann Arbor, MI, USA). 1-*O*-Hexadecyl-2-deoxy-2-amino-*sn*-glycerol (**1**) was obtained from Bachem AG (Bubendorf, Switzerland). Compound (**1**) is also available from SantaCruz Biotechnology (Dallas, TX, USA) or can be synthesized from L‑serine methyl ester (abcr GmbH, Karlsruhe, Germany) using a published method [20]. Chloroform, ethanol, hexane, ethyl acetate, isopropanol, and methanol (all of analytical grade) were sourced from VWR International (Radnor, PA, USA). Synthetic phospholipids were dissolved in chloroform and stored at –70 °C. Phospholipid concentrations were determined by a phosphorus microassay [21].

### Thin layer chromatography

Thin layer chromatography (TLC) was performed on Kieselgel 60 TLC plates (VWR International) using the following solvents: (**A**) chloroform-methanol (10:1, v/v), (**B**) hexane-ethyl acetate-triethylamine (10:4:0.5 v/v/v), or (**C**) chloroform-methanol-water (100:50:10, v/v/v). After developing of chromatograms, the plates were immersed in a 10 % CuSO_4_/8.5 % H_3_PO_4_ solution and subsequently charred. To detect compound (**3**), TLC plates were immersed in hexane-TEA (10:0.5 v/v) immediately before sample application to prevent the decomposition of phosphine (**3**) on the silica surface.

### Mass-spectrometric analysis of synthesized compounds

Mass spectra of synthetic compounds were recorded on an LTQ-XL ion-trap mass-spectrometer at a voltage of 1.9 kV, a capillary temperature of 200 °C, and a capillary voltage of 45V. The samples were dissolved in 80 % methanol (MeOH) supplemented with 1 % (v/v) formic acid (Fluka, St. Louis, MO, USA). Spectra of synthetic phospholipids were recorded in positive ion mode, with *m/z* values ranging from 100 to 2000 (or between 200 and 2000).

### NMR analysis of synthesized compounds

^1^H NMR and ^31^P-NMR spectra were recorded on a Varian Unity Inova 400 (300 K) or a Bruker AVANCE III 300 spectrometer, respectively. Chemical shifts (*δ*) are reported in ppm relative to tetramethylsilane as the standard.

### Chemical syntheses

#### 1-O-hexadecyl-2-deoxy-2-(tert‑butoxycarbonyl)amino-sn-glycerol (**2**)

To a solution of 1-*O*-hexadecyl-2-deoxy-2-amino-*sn*-glycerol (**1**) (32.0 mg, 101.4 µmol) in 3 ml of anhydrous dichloromethane, 26.16 µl of Boc anhydride (111.6 µmol) and 10 µl of TEA were added. The reaction was allowed to proceed for 4 h, after which it was complete, as confirmed by TLC analysis. The mixture was then evaporated under a stream of argon. The product (**2**) was purified by silica gel column chromatography, eluting with a gradient from 0 % to 5 % of MeOH in chloroform (v/v). Yield: 41.2 mg (98.0 %). R_f_ 0.81 (system A). ^1^H NMR (CDCl_3_) (δ, ppm): 0.88 (t, 3H, *J* = 6.6 Hz), 1.22–1.38 (m, 26H), 1.46 (s, 9 H), 1.55 (m, 2H), 2.74 (broad s, 1H), 3.43 (t, 2H, *J* = 6.6 Hz), 3.59 (m, 2H), 3.64 (m, 1H), 3.80 (m, 2H), 5.19 (broad s, 1H).

#### 1-O-hexadecyl-2-deoxy-2-(tert‑butoxycarbonyl)amino-sn‑glycero-3-(1-(N,N-diisopropylamino)-2-cyanoethyl)-phosphine (**3**)

Compound (**2**) (42 mg, 0.101 mmol) was phosphitylated to the corresponding phosphine (**3**) using 2-cyanoethyl-*N,N,N*′,*N*′-tetraisopropyl phosphorodiamidite (64.2 µl, 0.202 mmol) and diisopropylammonium 1*H*-tetrazolide (11.5 mg, 0.067 mmol) in anhydrous dichloromethane (6.2 ml). After 2 h, TLC analysis indicated that the reaction was complete. Methanol (1 ml) was added to quench the reaction, and the product (**3**) was extracted by dichloromethane containing 5 % TEA (v/v) and washed several times with 10 % sodium bicarbonate. The organic phase was dried over sodium sulfate and evaporated to dryness. Phosphine (**3**) was then purified by column chromatography on a silica gel using a hexane-ethyl acetate-TEA (10:4:0.5 by vol.) mixure as the eluent [22,23]. Yield: 84.8 %. R_f_ 0.86 (system B). ^1^H NMR (CDCl_3_) (δ, ppm): 0.88 (t, 3H, *J* = 6.6 Hz), 1.17–1.38 (m, 40H), 1.44 (s, 9 H), 1.52 (m, 2H), 2.65 (m, 2H), 3.41 (m, 2H), 3.43 (m, 2H), 3.50–3.85 (m, 7H). ^31^P-NMR (CDCl_3_) (δ, ppm): 148.71 and 148.50.

#### 1-O-hexadecyl-2-deoxy-2-amino-sn‑glycero-3-phosphocholine trifluoroacetic acid salt (**6**)

Coupling of phosphoroamidite (**3**) with choline tosylate in the presence of 1*H*-tetrazole in anhydrous dichloromethane, followed by oxidation with *tert*‑butyl hydroperoxide and cleavage of cyanoethyl protective group with TEA, was performed as a TLC-monitored one-pot reaction [22]. Prior to the reaction, the rotary evaporator was pre-dried under vacuum for at least 2 h. The required amounts of (**3**) and choline tosylate were dissolved in 2–5 ml of anhydrous dichloromethane and evaporated. The dissolving and evaporating process was repeated two more times. The dry residue was then dissolved in anhydrous dichloromethane, and 1*H*-tetrazol was added. Once TLC analysis indicated that the reaction was complete, *tert*‑butyl hydroperoxide in dodecane was added as the oxidant, leading to the formation of triphosphate (**4**). After completion (as verified by TLC), the cyanoethyl protective group of (**4**) was removed by the addition of TEA. The partially protected diphosphate (**5**) was purified on a C18-SPE cartridge using a gradient of MeOH in water from 50 % to 100 % (v/v). All eluting solutions contained 0.2 % formic acid. Fractions containing pure phosphate (**5**) were evaporated under vacuum. The Boc protective group was then cleaved from (**5**) by adding excess trifluoroacetic acid in dichloromethane. Once the reaction was complete, the mixture was evaporated under vacuum, dissolved in chloroform-methanol (2:1, v/v), and used directly for subsequent reactions. The purity of compound (**6**) was confirmed by TLC, ^1^H NMR, and mass spectrometry. The concentration of compound (**6**) was determined using by phosphorus assay. Yield: 63.3 % (based on phosphoroamidite (**3**)). R_f_ 0.21 (system C). ^1^H NMR (CDCl_3_: CD_3_OD, 2:1, v/v) (δ, ppm): 0.88 (t, 3H), 1.22–1.38 (m, 24H), 1.37 (m, 2H), 1.5–1.62 (m, 2H), 3.36 (s, 9H), 3.58 (m, 1H), 3.72–3.81 (m, 4H), 3.9 (d, 2H), 4.24 (d, 4H). Calculated for C_24_H_54_N_2_O_5_P: 481.38 g/mol; found *m/z*: 481.5 [*M* + H]^+^.

#### 1-O-hexadecyl-2-deoxy-2-(prostaglandin E_2_)amino-sn‑glycero-3-phosphocholine (**9a**)

##### Variant A

In a dark glass vial with a screw cap and PFTE septum, 1 mg (2.95 µmol) of PGE_2_ was dissolved in 100 µl methyl acetate (or ethyl acetate). Then, 100 µl of 10 mg/ml solution of EDC (5.22 µmol) in dichloromethane, 44 µl of 10 mg/ml *N*‑hydroxy succinimide (3.80 µmol, dissolved in THF), and 1 ml of dichloromethane were added. The reaction mixture was overlaid with argon and kept in the dark at room temperature for 18 h. After the reaction was complete (as confirmed by TLC analysis), the product (PGE_2_-hydroxysuccinimide ester, (**8**)) was extracted using a method similar to the Folch procedure. Methanol (0.5 ml) was added and thoroughly mixed, followed by the addition of 0.375 ml of deionized water and extensive vortexing. The lower phase was transferred to a fresh glass vial and evaporated under a stream of argon at 37 °C. The remaining oily compound (**8**) was dissolved in 0.44 ml of THF and mixed with 1-*O*-hexadecyl-2-amino-2-deoxy-*sn*‑glycero-3-phosphocholine (**6**) (TFA salt, 1.76 mg, 2.95 µmol), which had been pre-dissolved in 1 ml of THF. Then, 10 µl of TEA were added. The reaction mixture was overlaid with argon and kept in the dark at 22 °C on a shaker for 18 h. TLC analysis (system C) indicated the presence of the product (**9a**). The mixture was evaporated under argon, and the product was purified on an SPE-C18 cartridge using a gradient from 50 % to 100 % methanol in water containing 0.2 % formic acid. The fractions containing the pure product (**9a**) were evaporated under a stream of argon. Yield: 2.0 mg (87 %). R_f_ 0.28 (system C). Calculated for C_44_H_84_N_2_O_9_P: 815.59 g/mol; found *m/z*: 815.67 [*M* + H]^+^.

##### Variant B

Prostangandin E_2_ residue from the compound (**8**) was almost completely incorporated into a phospholipid (**9**) when compound (**6**) was used in ≥ 2-fold excess over compound (**8**), and the reaction was conducted in 10 % MeOH in THF. To this end, 2.07 µl of a 48.25 mg/ml solution of (**6**) in chloroform-methanol (2:1, v/v), equivalent to 100 µg (0.168 µmol) of TFA salt of (**6**), were placed into a dark glass screw vial with a cap and PFTE-septum. The solvents were evaporated under a stream of argon, and the residue was dissolved in 170 µl of 10 % MeOH/THF (v/v). Next, 15 µl of a 10 % TEA solution dissolved in 10 % MeOH/THF (v/v) and 11.6 µl (37.7 µg, 0.0839 µmol) of a of 3.25 mg/ml solution of *N*‑hydroxy succinimide ester of PGE_2_ (**8**) in 10 % MeOH/THF were added. The reaction mixture was overlayed with argon and left in the dark on a shaker at 22 °C for 18 h. The mixture was then evaporated under a stream of argon. Isolation of the product (**9a**) was carried out as described in Variant A.

#### 1-O-hexadecyl-2-deoxy-2-(8-isoprostaglandin E_2_)amino-sn‑glycero-3-phosphocholine (**9b**)

The synthesis starting from 8-isoprostaglandin-E_2_ was performed following the procedure described for (**9a**). R_f_ 0.28 (system C). Calculated for C_44_H_84_N_2_O_9_P: 815.59 g/mol; found *m/z*: 815.67 [*M* + H]^+^.

#### 1-O-hexadecyl-2-deoxy-2-(prostaglandin A_2_)amino-sn‑glycero-3-phosphocholine (**9c**)

"The synthesis and purification were carried out as described for (**9a**)."R_f_ 0.35 (system C). Calculated for C_44_H_82_N_2_O_8_P: 797.58 g/mol; found *m/z*: 797.87 [*M* + H]^+^.

#### 1-O-hexadecyl-2-deoxy-2-(8-isoprostaglandin A_2_)amino-sn‑glycero-3-phosphocholine (**9d**)

"The synthesis and purification were carried out as described for (**9a**)."R_f_ 0.35 (system C). Calculated for C_44_H_82_N_2_O_8_P: 797.58 g/mol; found *m/z*: 797.54 [*M* + H]^+^.

#### 1-O-hexadecyl-2-deoxy-2-(prostaglandin F_2α_)amino-sn‑glycero-3-phosphocholine (**9e**)

The synthesis was carried out as described for (**9a**). Purification using ann SPE-C18 cartridge with a gradient of ethanol in water from 50 % to 100 % yielded the product (**9e**). R_f_ 0.21 (system C). Calculated for C_44_H_86_N_2_O_9_P: 817.61 g/mol; found *m/z*: 817.83 [*M* + H]^+^.

#### 1-O-hexadecyl-2-deoxy-2-(8‑hydroxy-octanoyl)amino-sn‑glycero-3-phosphocholine (**9f**)

The synthesis and purification starting from 8‑hydroxy-octanoic acid were carried out as described for (**9a**). R_f_ 0.20 (system C). Calculated for C_32_H_68_N_2_O_7_P: 623.48 g/mol; found *m/z*: 623.58 [*M* + H]^+^.

#### 1-O-hexadecyl-2-deoxy-2-(12‑hydroxy-dodecanoyl)amino-sn‑glycero-3-phosphocholine (**9g**)

The synthesis and purification starting from 12‑hydroxy-dodecanoic acid were performed as described for (**9a**). R_f_ 0.21 (system C). Calculated for C_36_H_76_N_2_O_7_P: 679.54 g/mol; found *m/z*: 679.58 [*M* + H]^+^.

## Description of the method

**Fig. 1:**
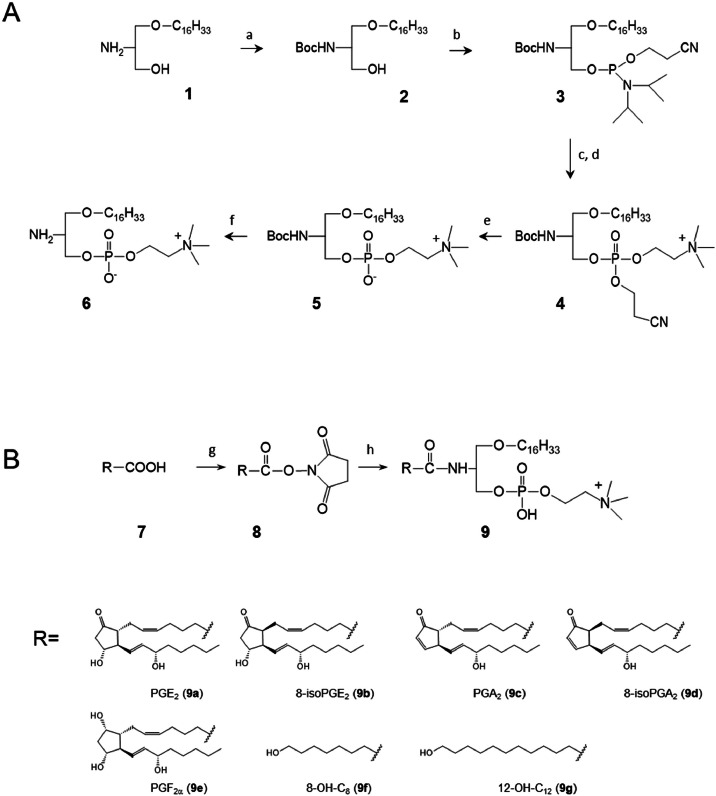
Scheme for the synthesis of alkyl-amide-PLs. (A) Synthesis of a common precursor 1-*O*-hexadecyl-2-amino-2-deoxy-*sn*‑glycero-3-phosphocholine (**6**). Reagents**: (**a) Boc_2_O, TEA; (b) [(isoPr)_2_N]_2_POCH_2_CH_2_CN, (isoPr)_2_NH salt of 1*H*-tetrazole, CH_2_Cl_2_; (c) choline tosylate, 1*H*-tetrazole; (d) *tert*‑BuOOH; (e) TEA; (f) TFA in CH_2_Cl_2_. (B) General scheme for the synthesis of alkyl-amide-PLs. Reagents: **(**g) oxylipin containing one carboxylic group, EDC, CH_2_Cl_2_; (h) lyso-amino-phospholipid (**6**), THF, TEA.

The synthesis of alkyl-amide-OxPCs was carried out in two stages. First, a common precursor for all alkyl-amide oxidized phosphatidylcholines, namely lyso-amino-PC (**6**), was synthesized. To prepare common precursor (**6**), six reaction steps were required, three of which were performed sequentially in a one pot process (Fig. 1A).

The starting material for the synthesis of (**6**) was 2-amino-2-deoxy-1-*O*-hexadecyl-*sn*-glycerol (**1**), the free amino group of which was protected by reaction with Boc anhydride to form compound (**2**). This compound was then phosphitylated using bis(diisopropylamino)-cyanoethoxyphosphine in dichloromethane under anhydrous conditions, in the presence of diisopropylammonium tetrazolide. The phosphitylation reaction proceeded quantitatively, as confirmed by TLC. The absence of residual water in both the reactants and solvent was essential for the reaction's success. Product (**3**) was isolated by extraction followed by chromatographic purification on a silica gel column. The solvent-free compound (**3**) could be stored without loss of activity for at least six months under an argon atmosphere at −80 °C.

The next three synthetic steps were carried out as a one pot reaction under the TLC monitoring. The reaction of compound (**3**) with choline tosylate was performed in anhydrous dichloromethane, with 1*H*-tetrazol acting as an activator. Once TLC indicated the reaction was complete, *tert*‑butyl hydroperoxide in dodecane was added as the oxidant, resulting in the formation of triphosphate (**4**). After completion of the reaction (verified by TLC), the cyanoethyl protective group of (**4**) was removed by adding TEA. The partially protected diphosphate (**5**) was isolated using solid-phase extraction (SPE), and the Boc- protecting group was removed by adding trifluoroacetic acid, which was then evaporated under reduced pressure. The final product (**6**) was then used without further purification. However, compound (**6**) could be further purified by SPE or column chromatography on C18-modified silica gel.

**Fig. 2:**
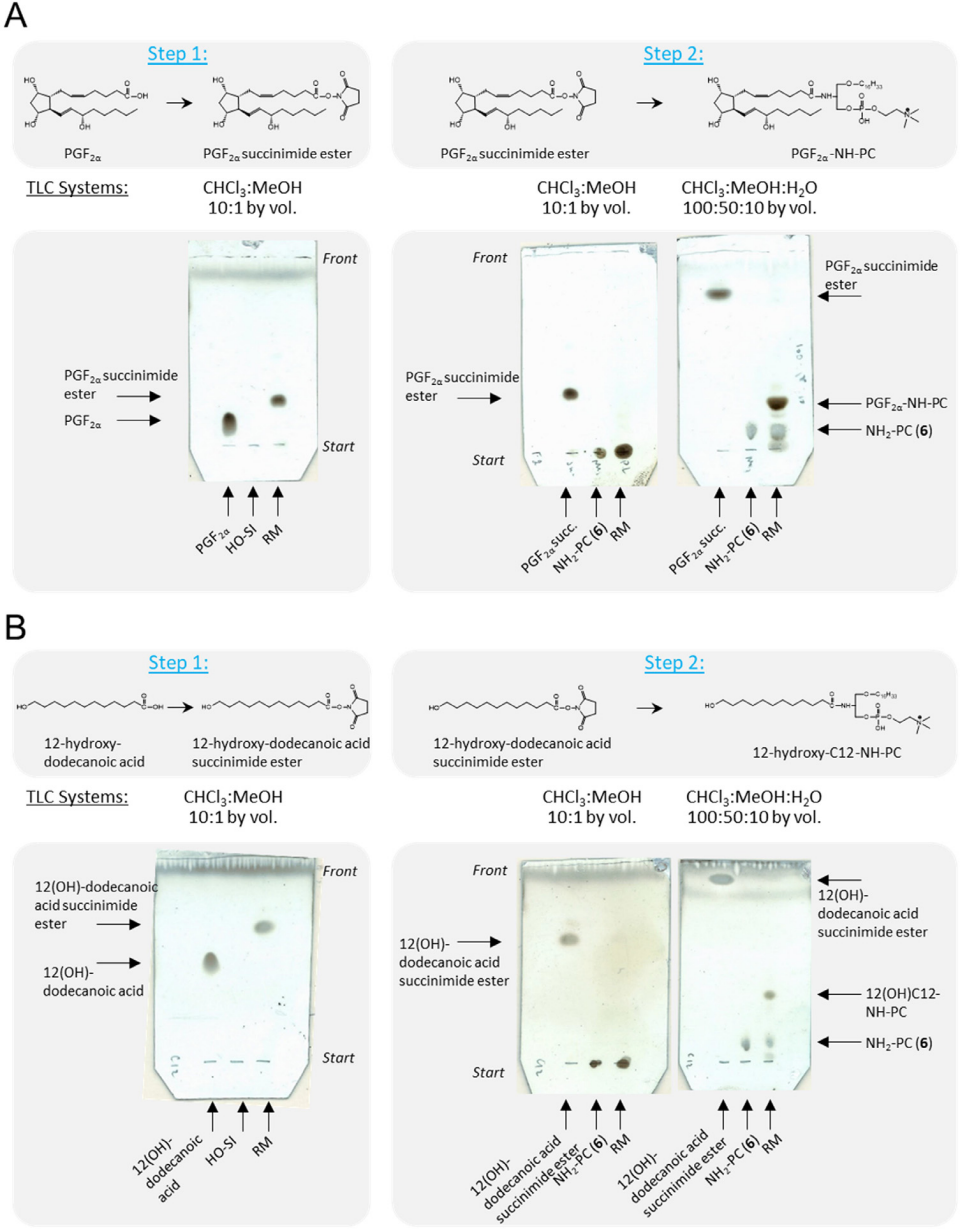
Examples of incorporation of oxylipins into glycerophospholipid scaffold. Prostaglandin F_2α_ (A) or 12‑hydroxy-dodecanoic acid (B) were activated by formation of hydrosuccinimide esters. TLCs of reaction mixtures (RM) illustrate full conversion of starting oxylipins (Step 1). Second educt (N-hydroxysuccinimide, HO-SI) is not visible under TLC conditions used. Succinimide esters of prostaglandin F_2α_ (A) or 12‑hydroxy-dodecanoic acid (B) were purified by liquid-liqid extraction and then reacted with 2-amino-2-deoxy-PC (NH_2_-PC, **6**). TLC analysis (Step 2) demonstrates complete incorporation of oxylipins.

During the second stage of alkyl-amide-OxPL synthesis (Fig. 1B), oxylipins were attached to the lyso-amino-PC scaffold (**6**) through two reactions. First, an oxylipin (**7**) was treated with N-hydroxysuccinimide in dichloromethane in presence of EDC as an activator, resulting in the formation of the activated ester (**8**). This reaction was characterized by complete conversion of the oxylipin (Fig. 2, Step 1). The resulting N-hydroxysuccinimide ester of the oxylipin (**8**) was isolated by liquid-liquid extraction using MeOH/dichloromethane/water mixture. During this extraction, unreacted EDC and N-hydroxysuccinimide were removed, as they were soluble in the water phase. The solvent of the organic phare containing the pure compound (**8**) was then evaporated under a stream of argon. In the second reaction, the activated ester (**8**) from the previous stage was reacted with lyso-amino-PC (**6**) in the presence of triethylamine. The reaction mixture was subsequently evaporated under a stream of argon, and the product (**9**) was isolated using a reversed-phase SPE-cartridge.

This method was successfully applied to several prostanoid oxylipins, as well as linear oxylipins containing ω-terminal hydroxyl groups (Fig. 1, Fig. 2). The oxylipin-containing products **9a-9d** and **9f-9g** were purified by C18-SPE using a gradient of 70 % to 100 % methanol in water. Some oxylipins required different solvents for elution from the SPE columns, depending on the polarity of the product (**9**). For example, prostaglandin F2α-containing phosphatidycholine (**9e**) was eluted using a gradient of ethanol in water.

**Fig. 3:**
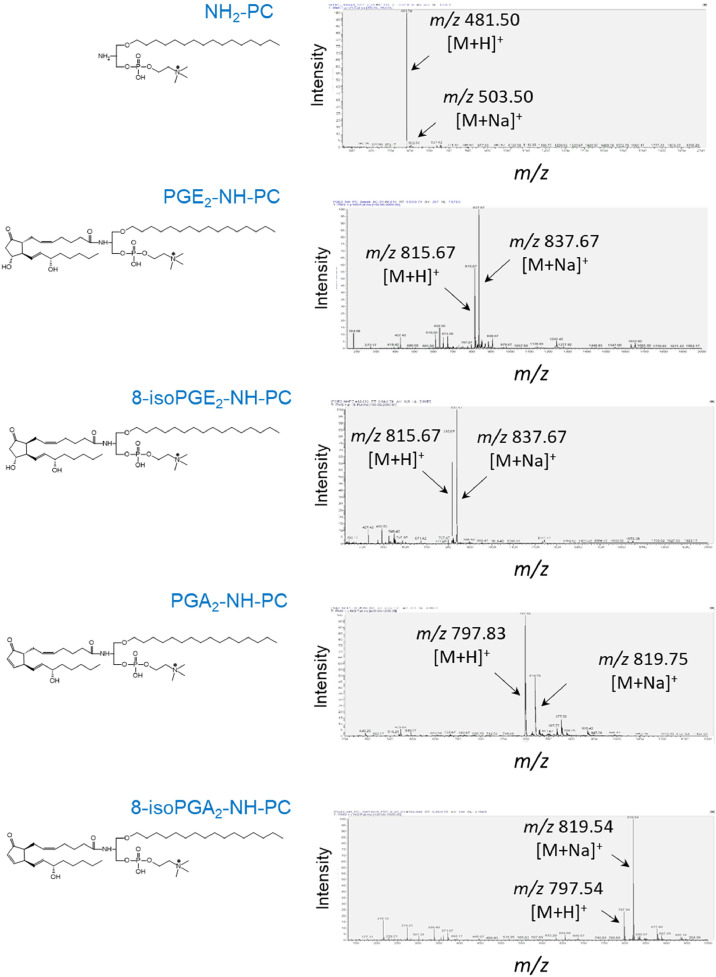

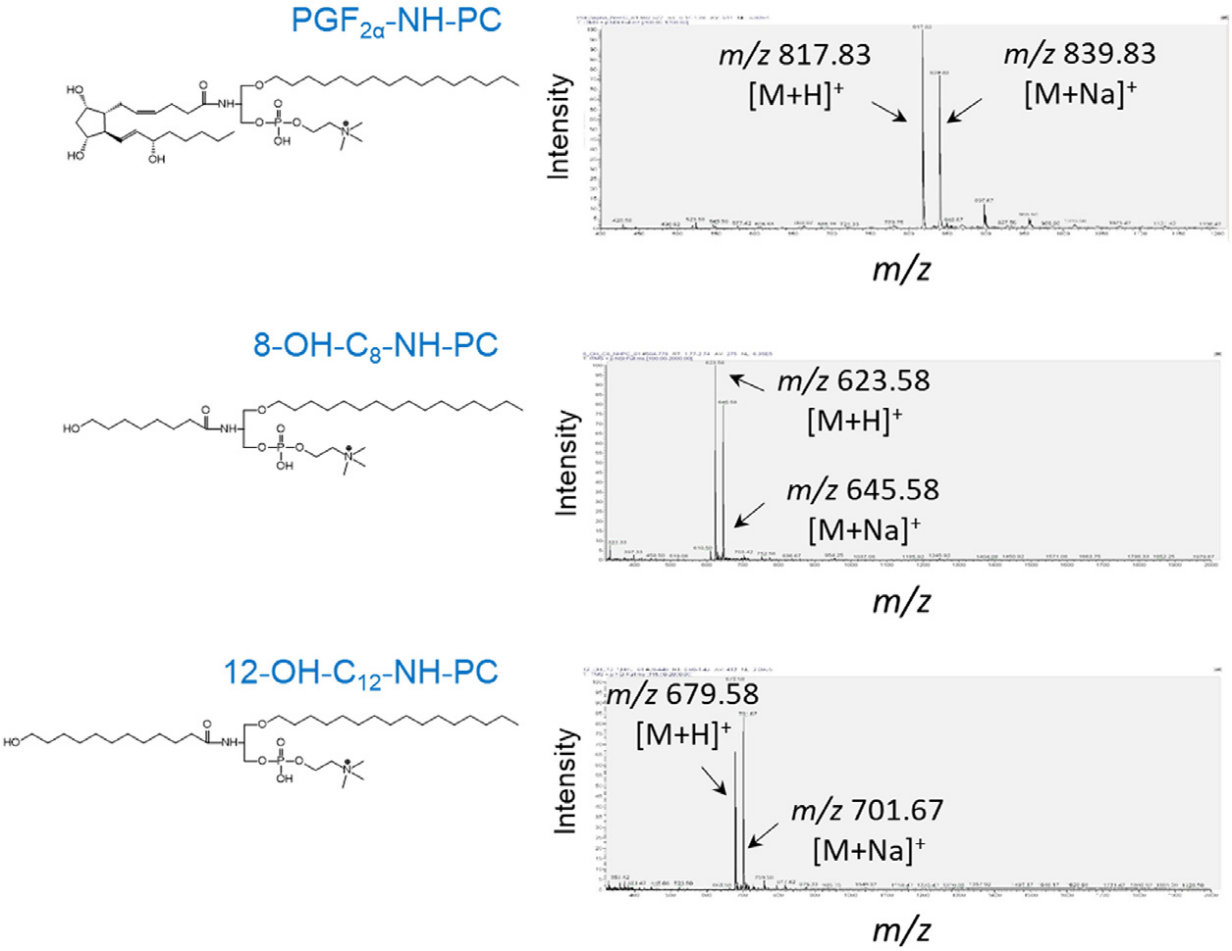
Mass spectra of synthetic phospholipids were recorded in the positive ion mode.

The influence of different solvents on the final coupling reaction, using PGE_2_ as a sample oxylipin was tested. It was found that the coupling of PGE_2_ to the phospholipid scaffold yielded comparable results in various solvents, including tetrahydrofuran-dichloromethane (1:1 or 2:1, v/v), tetrahydrofuran-methanol (10:1, v/v), tetrahydrofuran-acetonitrile-methanol (3:1:0.5, v/v/v), and in the presence of water (up to 10 % volume in THF) or tertiary amines (up to 10 % TEA, v/v). In all cases, the yield of the final coupling reaction, using equimolar amounts of oxylipins and lyso-amino-PC (**6**), was approximately 50 %. However, the addition of a 2-fold molar excess of lyso-amino-PC (**6**) resulted in nearly complete incorporation of oxylipins into phospholipids (Fig. 1B, Fig. 2A Step 2B, and Fig. 2B, Step 2). The synthesized phospholipid compounds were analyzed for purity using TLC and mass spectrometry (Fig. 3). The amounts of compounds (**6)** and (**9a-9g**) were determined by phosphorus microassay [21].

## Conclusions

In this work, a versatile approach for the chemical synthesis of multiple oxidized phospholipids with the alkyl-amide-scaffold is described. This method allows for the fast and efficient incorporation of oxylipins, such as (iso)prostanes, into the phospholipid scaffold without the need to protect/deprotect functional groups. This is a significant advantage, as oxylipins - especially (iso)prostanes - are prone to chemical degradation during protection/deprotection cycles. Several compounds can be prepared within a few days in amounts sufficient to perform biological assays *in vitro* and *in vivo*. The results demonstrate the high potential of this method for preparing a wide variety of oxylipin-containing PLs using commercially available oxylipins, which would be valuable for screening of OxPLs in various biological tests.

## Method validation

Alkyl-amide phospholipids **9a-9g** were prepared with the goal of using them as anti-lipopolysaccharide (anti-LPS) and endothelial barrier-protecting compounds. It has been found that oxidized phospholipids with the alkyl-amide scaffold are effective anti-LPS compounds, demonstrating activity both *in vitro* and *in vivo* [19]. Additionally, they were able to enhance the endothelial barrier in quiescent lung endothelial cells and restore the barrier function disrupted by LPS, IL-6, or thrombin as tested in both *in vitro* and *in vivo* models [19]. In summary, the effects of alkyl-amide oxidized phospholipids were found to be identical to those induced by diacyl analogs, specifically oxidized 1-palmitoyl-2-arachidonoyl-*sn*‑glycero-3-phosphocholine [24,25].

## Limitations

The method described here enables the chemical synthesis of oxidized PLs with a choline head group. However, to synthesize PLs with different head group functionalities, the use of protective groups is required. Another limitation is that the method can only utilize oxylipins with a single carboxylic group. For the synthesis of OxPLs containing ω-terminal carboxylic groups in residues attached at the *sn*-2 position, the introduction of protective groups would be necessary.

## Ethics statements

N/A

## Declaration of generative AI and AI-assisted technologies in the writing process

During the preparation of this work, the author(s) used ChatGPT to check for correct syntax and spelling, as well as to enhance clarity and flow while maintaining the technical accuracy. After using this tool, the authors reviewed and edited the content as necessary. The authors take full responsibility for the content of the publication.

## CRediT authorship contribution statement

**Olga V. Oskolkova:** Conceptualization, Investigation, Visualization, Writing – original draft, Writing – review & editing, Project administration, Supervision. **Bernd Gesslbauer:** Investigation, Visualization, Funding acquisition. **Valery Bochkov:** Writing – original draft, Writing – review & editing, Supervision, Funding acquisition.

## Declaration of competing interest

The authors declare the following financial interests/personal relationships which may be considered as potential competing interests: OVO, AB, KB and VB are inventors on a patent related to work on OxPLs.

## Data Availability

No data was used for the research described in the article.
